# Prevalence, Risk Factors, Lung Function, and Associated Comorbidities of Adult Preserved Ratio Impaired Spirometry: A Meta‐Analysis

**DOI:** 10.1002/mco2.70235

**Published:** 2025-05-31

**Authors:** Haoyu Wang, Ruiyuan Yang, Dan Liu, Weimin Li

**Affiliations:** ^1^ Department of Pulmonary and Critical Care Medicine West China Hospital Sichuan University Chengdu Sichuan China; ^2^ State Key Laboratory of Respiratory Health and Multimorbidity West China Hospital Chengdu Sichuan China; ^3^ Precision Medicine Center Precision Medicine Key Laboratory of Sichuan Province West China Hospital Sichuan University Chengdu Sichuan China; ^4^ The Research Units of West China Chinese Academy of Medical Sciences West China Hospital Chengdu Sichuan China; ^5^ Institute of Respiratory Health Frontiers Science Center for Disease‐Related Molecular Network West China Hospital Sichuan University Chengdu Sichuan China

**Keywords:** preserved ratio impaired spirometry, prevalence, risk factor, comorbidities, meta‐analysis

## Abstract

It is demonstrated that preserved ratio impaired spirometry (PRISm) is associated with chronic obstructive pulmonary disease development and mortality. However, comprehensive evidence on its prevalence, risk factors, lung function, and comorbidities is ambiguous. We searched for relevant studies from Medline, Web of Science, Embase, and Scopus up to March 26, 2024, and conducted a meta‐analysis based on PRISMA 2020 to merge the results of eligible studies to reveal the prevalence, risk factors, lung function, and associated comorbidities in PRISm population. Thirty‐two studies involving 1,196,856 participants were included. The prevalence of PRISm was 11% (95%CI: 10–13%), with decreased forced vital capacity (FVC) (L) (MD: −0.78, 95%CI: −0.90 to −0.66) and FVC% predicted (MD: −24.74, 95%CI: −26.33 to −23.16). Older age, high body mass index, current or ever smoking, and low education were positively associated with PRISm, while cardiovascular and endocrine comorbidities were common in patients with PRISm. The prevalence of PRISm is high in general population, with multiple risk factors, reduced lung function, and increased comorbidities. Therefore, clinicians should raise more concerns regarding this population to benefit them.

## Graphical abstract

Comprehensive evidence on PRIsm prevalence, risk factors, lung function, and comorbidities is limited. We performed a meta‐analysis of 32 studies with 1,196,856 participants and found PRISm prevalence at 11%, with risk factors like age, body mass index, smoking, and education. Cardiovascular and endocrine comorbidities are common in PRISm. Therefore, clinicians should be more aware of this population for better patient care

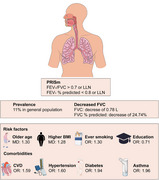

## 1 Introduction

Chronic obstructive pulmonary disease (COPD) ranks as one of the most common respiratory diseases with increasing morbidity and mortality, leading to severe disease burden, poor quality of life, and high treatment expenses [1, 2]. Although COPD is not curable, symptoms and adverse outcomes can be controlled early and even prevented. Consequently, the identification of impaired lung function or early COPD has become increasingly concerned.

A substantial proportion of individuals characterized by a preserved forced expiratory volume in 1 s (FEV_1_)/forced vital capacity (FVC) ratio (≥0.7) and a reduced FEV_1_% of the predicted value (<0.8), which used to be an unclassified or nonspecific pattern, has recently been clarified as preserved ratio impaired spirometry (PRISm) by Global Initiative for Chronic Obstructive Lung Disease (GOLD), suggesting its potential role as an early form of abnormal phenotype associated with COPD development [3, 4, 5].

However, previous evidence indicated that patients with PRISm are a heterogeneous population under a transitional status with an uncertain trajectory, as approximately 25.1% of PRISm cases may develop airflow obstruction or COPD, while 22.2% may revert to normal [6]. Furthermore, it is revealed that PRISm is strongly associated with an elevated risk of comorbidities and mortality, especially in smokers [6, 7, 8, 9]. Therefore, PRISm may not be simply regarded as the early stage of COPD due to its transitional nature and potential adverse outcomes, and increased attention is warranted to facilitate early identification and intervention in the PRISm population, especially for those with comorbidities or a high risk of adverse outcomes.

Previous studies have demonstrated that PRISm is associated with mortality in general population. However, comprehensive evidence on the epidemiology of PRISm is lacking. Therefore, we conducted this meta‐analysis to investigate the prevalence, risk factors, lung function, and comorbidities in PRISm population. Our findings revealed that the prevalence of PRISm was 11% in general population, with multiple risk factors like age, BMI, smoking status, and education, and reduced lung function. Moreover, cardiovascular, metabolic and endocrine comorbidities are common in patients with PRISm. Our study provided an overview of this phenotype for physicians in clinical settings, which may assist in identification and treatment at an earlier phase.

## 2 Results

### 2.1 Literature Selection

**FIGURE 1:**
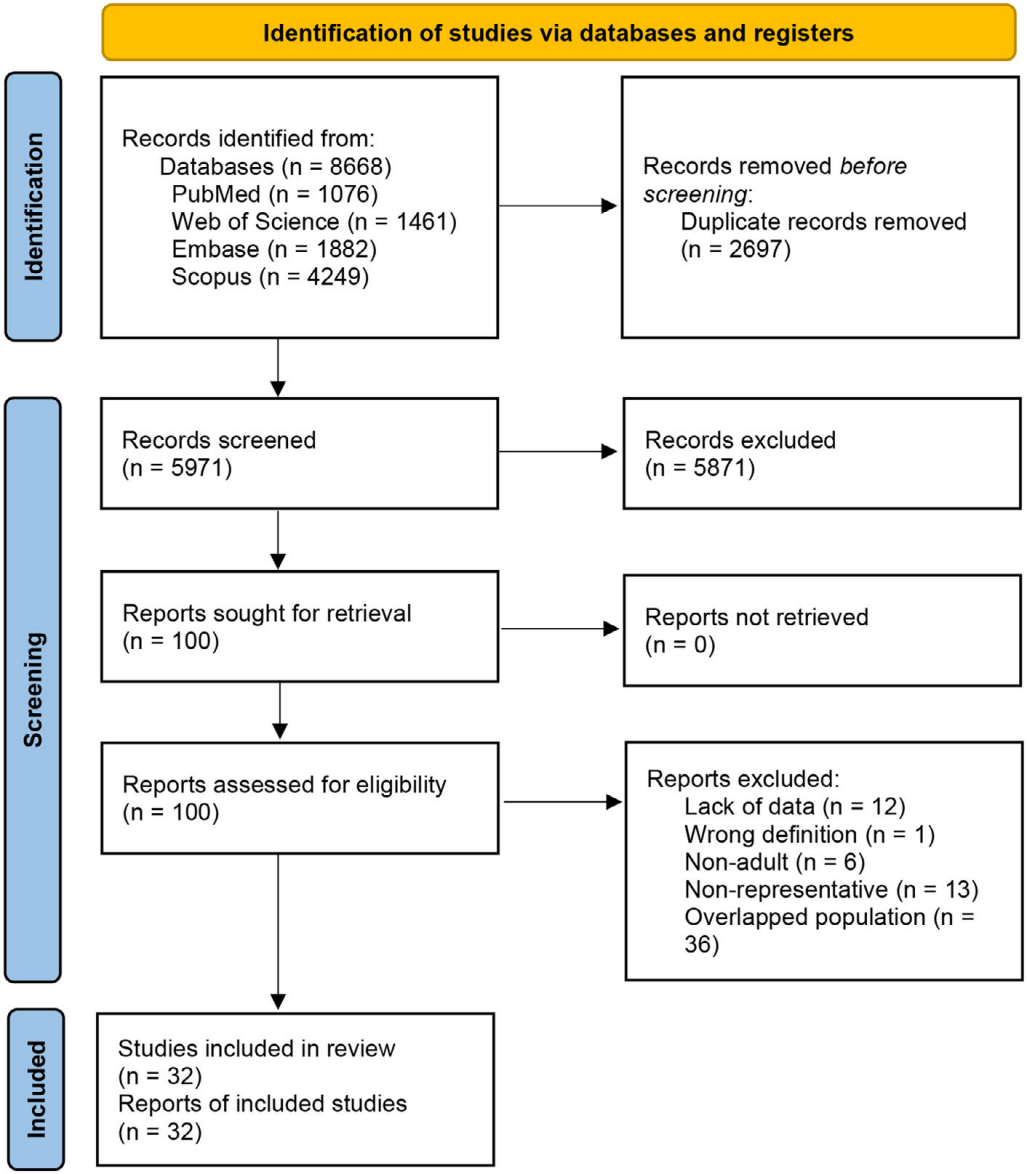
PRISMA flow diagram of this study.

A total of 8668 records were initially retrieved from electronic databases, and 2697 duplicates and 5871 irrelevant studies were removed before full‐text review (Figure 1). Of the 100 studies comprehensively reviewed, only 32 studies involving 1,196,856 participants were ultimately included in the meta‐analysis [10, 11, 12, 13, 14, 15, 16, 17, 18, 19, 20, 21, 22, 23, 24, 25, 26, 27, 28, 29, 30, 31, 32, 33, 34, 35, 36, 37, 38, 39, 40, 41].

### 2.2 Main Characteristics

The main characteristics of included studies were displayed in Table 1. Most studies were performed in general population using prebronchodilator spirometry and the fixed ratio definition, while two studies focused on smokers. Besides, more than half of the studies were conducted in Asia, while others were in Europe and America. All studies included adult participants, most of whom were over 40 years old. The Newcastle‐Ottawa Quality Assessment Scale (NOS) ranged from 6 to 9 points, suggesting the high quality of included studies. The detailed NOS assessment was presented in Table S1.

**TABLE 1:** Main characteristics of included studies.

Author year	Study/cohort	Region	Population	Subject	Case	Age	Definition	Pre/Post‐BD	Nos.
Wijnant 2020	Rotterdam study	Netherlands	General population	5487	387	≥45	Fixed ratio	Pre	7
Anami 2021	NR	Japan	General population	668	80	≥60	Fixed ratio	Post	7
Kaise 2021	OCEAN study	Japan	General population	2518	420	≥40	Fixed ratio	Pre	6
Marott 2021	Copenhagen city heart study	Denmark	General population	2387	619	20–40	Fixed ratio	Pre	7
Schwartz 2021	NR	USA	General population	6494	1342	>18	LLN	Pre	6
Wan 2021	NHLBI pooled cohorts study	USA	General population	53,701	4582	≥18	Fixed ratio	Pre	8
Higbee 2022	UK Biobank	UK	General population	35,1874	38,639	40–69	Fixed ratio	Pre	8
Kaaks 2022	LUSI	German	Smokers in lung cancer screening	1987	311	50–69	Fixed ratio	Pre	8
Kanetake 2022	NR	Japan	General population	1672	176	56.5 ± 9.5^a^	LLN	Pre	8
Kim 2022	KNHANES	Korea	General population	17,515	1563	≥50	Fixed ratio	NR	8
Shiraishi 2022	NR	Japan	Lung cancer screening	1818	173	≥40	Fixed ratio	Pre	7
Tanabe 2022	NR	Japan	General population	10,828	706	≥40	Fixed ratio	Pre	8
Tang 2022	The Enjoying Breathing Program	China	General population	32,033	6325	≥18	Fixed ratio	Pre	6
Washio 2022	Hisayama study	Japan	General population	3032	301	≥40	Fixed ratio	Pre	8
Zhao 2022	ECOPD	China	General population	1439	126	40–80	Fixed ratio	Post	6
Chen 2023	NHANES	USA	General population	9556	671	18–79	Fixed ratio	Pre	8
Fan 2023	National cross‐sectional survey of COPD surveillance	China	General population	3526	243	≥40	Fixed ratio	Post	8
Kogo 2023	Nagahama study	Japan	General population	9760	438	30–74	LLN	Pre	9
Krishnan 2023	CanCOLD	Canada	General population	1561	96	67 ± 10^a^	Fixed ratio	Post	8
Labaki 2023	COPDGene	USA	Smokers in general population	10,132	1262	45–80	Fixed ratio	Post	8
Magner 2023	UCAP	Canada	General population	2514	169	≥18	Fixed ratio	Post	6
Marott 2023	Copenhagen general population study	Denmark	General population	106,845	6128	20–100	LLN	Pre	8
Miura 2023	NR	Japan	General population	11,246	838	35–65	Fixed ratio	Pre	6
Perez‐Padilla 2023	PLATINO	Mexico, Venezuela, Brazil, Uruguay, and Chile	General population	2942	146	≥40	Fixed ratio	Post	8
Sin 2023	KoGES	Korea	General population	7526	471	40–69	Fixed ratio	Pre	7
Zhang 2023	PIFCOPD	China	General population	1183	221	40–75	Fixed ratio	NR	7
Choi 2024	KOCOSS	Korea	General population	123	21	61 (52–67)^a^	Fixed ratio	Post	8
He 2024	ELSA	UK	General population	5901	817	≥50	Fixed ratio	Pre	7
lm 2024	NR	Korea	General population	11,420	2884	≥40	Fixed ratio	Pre	7
Lei 2024	CPH study	China	General population	50,991	2459	≥20	Fixed ratio	Post	6
Shu 2024	NR	Taiwan, China	General population	461,183	65,832	≥20	Fixed ratio	Pre	8
Zhang 2024	NR	China	General population	6994	1997	35–70	Fixed ratio	Pre	7

Abbreviations: BD: bronchodilator; CanCOLD: Canadian cohort obstructive lung disease; COPDGene: genetic epidemiology of chronic obstructive pulmonary disease; CPH: China pulmonary health; ECOPD: early chronic obstructive pulmonary disease; ELSA: English longitudinal study of ageing; KNHANES: Korea National Health and Nutrition Examination Survey; KOCOSS: Korean Chronic Obstructive Pulmonary Disorders Subgroup Study; KoGES: Korean Genome and Epidemiology Study; LLN: lower limit of normal; LUSI: Lung Cancer Screening Intervention Study; NHANES: National Health and Nutrition Examination Assessment; NHLBI: National Heart, Lung, and Blood Institute; NOS: Newcastle‐Ottawa Quality Assessment Scale; NR: not reported; OCEAN: Okinawa COPD case finding assessment; PIFCOPD: predictive value of combining inflammatory biomarkers and rapid decline of FEV1 for chronic obstructive pulmonary disease; PLATINO: The Proyecto Latinoamericano de Investigación en Obstrucción Pulmonary; UCAP: undiagnosed chronic obstructive lung disease and asthma population; UK: United Kingdom; USA: United States of America.

^a^ Reported as mean ± standard deviation or median (interquartile range). Others were reported as range.

### 2.3 Prevalence of PRISm

**FIGURE 2:**
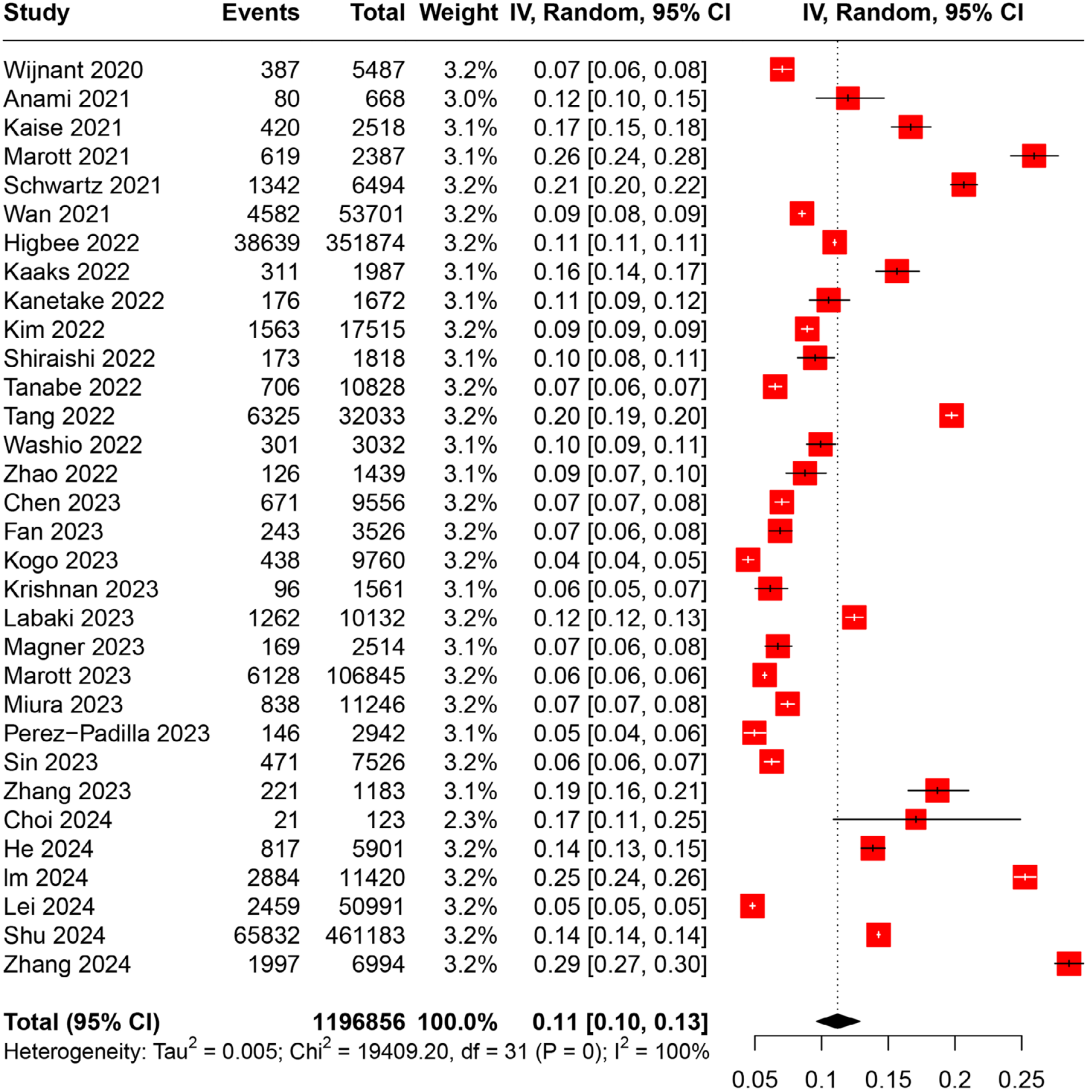
Forest plot of the prevalence of PRISm. IV: inverse variance; CI: confidence interval.

**FIGURE 3:**
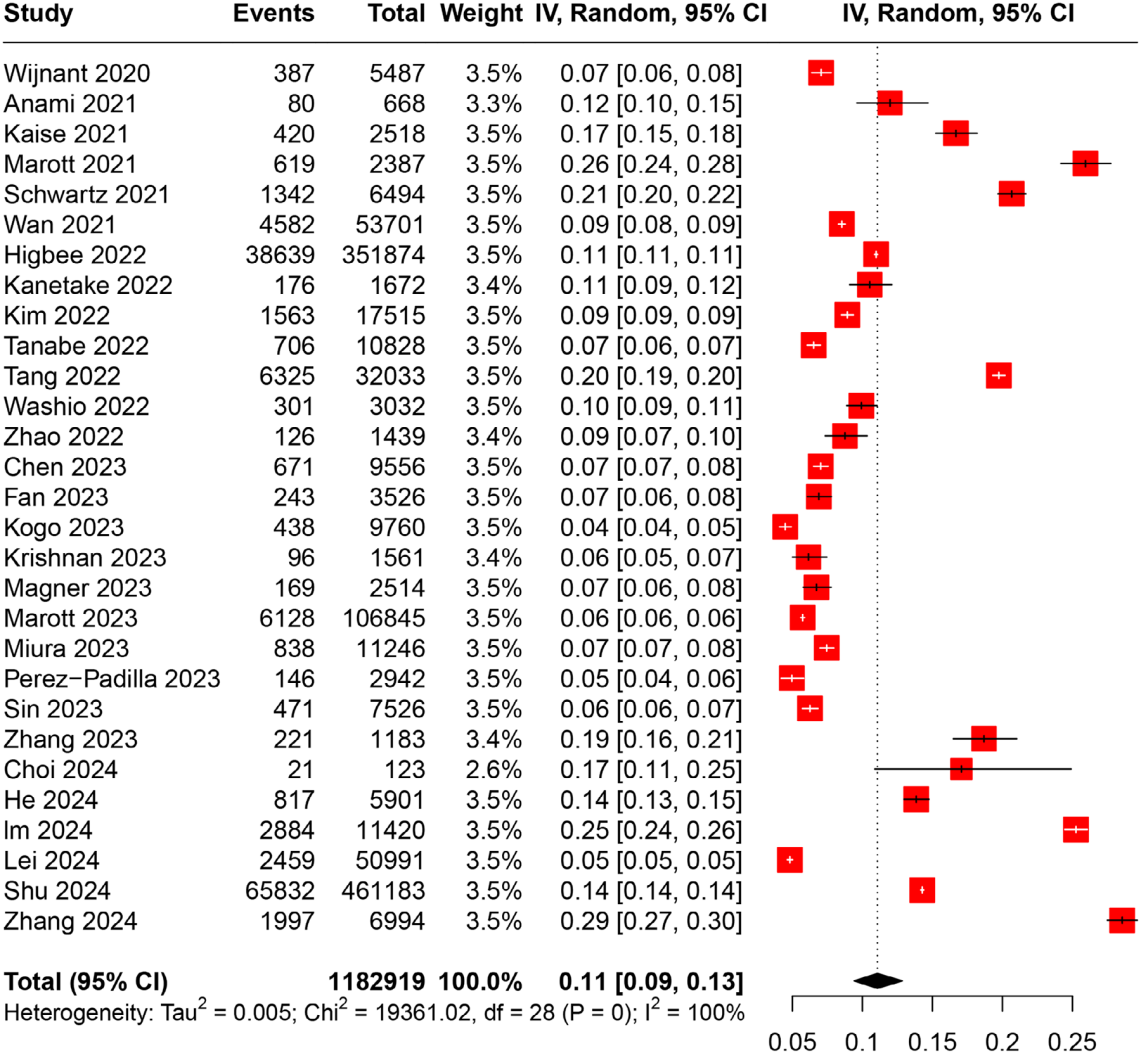
Sensitivity analysis of the prevalence of PRISm by including only general population. IV: inverse variance; CI: confidence interval.

In the overall population, the prevalence of PRISm was 11% (95%CI: 10–13%), with high heterogeneity (*I* ^2^ = 100%, *p* < 0.01) (Figure 2). Sensitivity analysis by excluding studies based only on smokers or lung cancer screening participants revealed the robustness of the results, but no decrease in heterogeneity was observed (pooled prevalence: 11%, 95%CI: 9–13%, *I* ^2^ = 100%, *p* < 0.01) (Figure 3). Leave‐one‐out sensitivity analysis further confirmed the robustness of pooled results (Figure S1). Further stratification by sex revealed the prevalence of PRISm was similar in males and females (males: 11%, 95%CI: 10–13%; females: 11%, 95%CI: 9–13%) (Table S2). Moreover, stratification by smoking status revealed a potential trend in PRISm prevalence with changes of smoking status (never smoker: 10%, 95%CI: 8–12%; ever smoker: 11%, 95%CI: 10–13%; current smoker: 13%, 95%CI: 11–14%) (Table S2). Subgroup analyses based on PRISm definition, pre‐ or postbronchodilator, study region, sample size, and NOS showed that the prevalences of PRISm in lower limits of normal (LLN), postbronchodilator, and American subgroups were mildly lower but not significantly different (Table S2). However, when comparing subgroups of prebronchodilator and postbronchodilator measurements alone, the results are significant (prebronchodilator: 12%, 95%CI: 10–14%; postbronchodilator: 8%, 95%CI: 6–11%; *p* = 0.02), suggesting that the use of bronchodilator might contribute to heterogeneity. Besides, studies with an NOS score of 8–9 were significantly lower than those with a score of 6–7 (8–9: 9%, 95%CI: 7–11%; 6–7: 14%, 95%CI: 9–19%; *p* = 0.04), indicating the need for future high‐quality studies to validate these results.

To detect the potential contributors of heterogeneity, we then conducted meta‐regression. The results showed that the definition of PRISm may account for 13.1% of heterogeneity, but without significance (*p* = 0.40) (Table 2). Relatively, the NOS showed a significant trend but the *R* ^2^ statistics were few (Table 2). These two factors may contribute to heterogeneity but with limited effect. Therefore, the high heterogeneity may also be caused by other factors of included cohorts.

**TABLE 2:** Meta‐regression for the prevalence of PRISm.

	Regression coefficients		Amount of accounted or residual heterogeneity				Test for residual heterogeneity		Test of moderators	
Factor	Estimate	*p*	Tau [2]	*I* ^2^ (%)	*H* ^2^	*R* ^2^ (%)	QE	*p*	QM	*p*
Definition			0.0047	99.8	489.87	13.1	14,696.07	<0.01	0.72	0.40
Fixed ratio	ref									
LLN	−0.03	0.40								
Region			0.0074	99.8	548.26	0	15,899.55	<0.01	1.67	0.43
America	ref									
Asia	0.04	0.26								
Europe	0.06	0.24								
Population			0.0054	99.9	646.46	0	19,393.91	<0.01	0.22	0.64
General population	ref									
Nongeneral population	0.02	0.64								
Sample size			0.0055	99.9	645.71	0	19,371.20	<0.01	0.19	0.66
≥10,000	ref									
<10,000	0.01	0.66								
NOS			0.0058	99.9	646.56	0	19,396.85	<0.01	7.51	<0.01
8–9	ref									
6–7	0.07	<0.01								

Abbreviations: LLN: lower limit of normal; NOS: Newcastle‐Ottawa Quality Assessment Scale.

**FIGURE 4:**
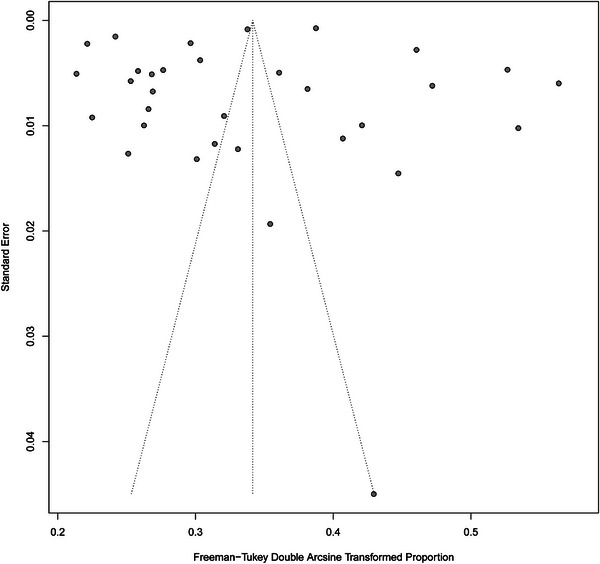
Funnel plot of the prevalence of PRISm.

Baujat plot suggested that the study by Marrot contributed the most heterogeneity (Figure S2), and funnel plot and Egger's test suggested that no obvious publication bias existed (*p* = 0.54) (Figure 4) [31].

### 2.4 Risk Factors of PRISm

As Table 3 showed, an elevated risk of PRISm was associated with older age (years) (MD: 1.30, 95%CI: 0.78–1.81), high BMI (MD: 0.89, 95%CI: 0.48–1.30), male sex (OR: 1.12, 95%CI: 1.02–1.23), ever smoking (OR: 1.30, 95%CI: 1.15–1.46), and current smoking (OR: 1.45, 95%CI: 1.19–1.76). Postsecondary education is associated with a reduced risk of PRISm (OR: 0.71, 95%CI: 0.54–0.93) compared with individuals with only secondary education or less. Egger's test indicated the publication bias of age and sex (age: *p* < 0.01; sex: *p* = 0.01), so we conducted trim and fill analyses. Interestingly, the effect direction of sex reversed after a trim and fill method (OR: 0.89, 95%CI: 0.81–0.98) (Table S3). As we formerly found the prevalence of PRISm may change with different smoking status (Table S2), we hypothesized that this effect of male sex might be confounded by factors such as smoking status, while age may also be a confounder. Nonetheless, whether females are truly at higher risk of developing PRISm requires further investigation.

**TABLE 3:** Risk factors, lung function, and associated comorbidities of PRISm.

	Number of studies	Measurement	Estimate (95% CI)	*p* ^a^	*I* ^2^ (%)	*p*_Cochrane_ ^b^	*p*_Egger_ ^c^
Risk factors							
Age (years)	23	MD	1.30 (0.78 to 1.81)	<0.01	99	<0.01	<0.01
BMI	21	MD	0.89 (0.48 to 1.30)	<0.01	100	<0.01	0.19
Sex (male)	29	OR	1.12 (1.02 to 1.23)	0.01	97	<0.01	0.01
Ever smoker	24	OR	1.30 (1.15 to 1.46)	<0.01	98	<0.01	0.09
Current smoker	25	OR	1.45 (1.19 to 1.76)	<0.01	99	<0.01	0.11
Postsecondary education	7	OR	0.71 (0.54 to 0.93)	0.01	97	<0.01	0.57
Lung function							
FVC (L)	12	MD	−0.78 (−0.90 to −0.66)	<0.01	99	<0.01	0.05
FVC% predicted	16	MD	−24.74 (−26.33 to −23.16)	<0.01	100	<0.01	0.02
Comorbidities							
Cardiovascular disease (overall)	9	OR	1.59 (1.31 to 1.92)	<0.01	50	0.04	0.14
Hypertension	16	OR	1.60 (1.29 to 2.00)	<0.01	99	<0.01	0.16
Ischemic heart disease	9	OR	1.98 (1.71 to 2.30)	<0.01	76	<0.01	0.19
Heart failure	5	OR	3.22 (1.90 to 5.47)	<0.01	90	<0.01	0.03
Stroke	8	OR	2.09 (1.78 to 2.44)	<0.01	50	0.05	0.50
Diabetes	20	OR	1.94 (1.71 to 2.19)	<0.01	95	<0.01	<0.01
Dyslipidemia	5	OR	1.34 (1.17 to 1.54)	<0.01	67	0.02	0.85
Obesity or overweight	4	OR	1.65 (1.16 to 2.34)	<0.01	93	<0.01	0.53
Osteoporosis	3	OR	1.16 (0.96 to 1.40)	0.12	0	0.84	0.60
Chronic bronchitis	5	OR	1.82 (1.47 to 2.24)	<0.01	63	0.03	0.20
Asthma	11	OR	1.96 (1.77 to 2.17)	<0.01	71	<0.01	0.77
Tuberculosis	4	OR	1.44 (0.99 to 2.10)	0.06	0	0.69	0.84
Cancer	5	OR	1.16 (0.91 to 1.47)	0.23	24	0.26	0.39
Chronic kidney disease	5	OR	1.66 (1.03 to 2.67)	0.04	98	<0.01	0.39
Anemia	3	OR	0.87 (0.61 to 1.25)	0.45	84	<0.01	0.12
Depression	2	OR	1.06 (0.96 to 1.17)	0.25	0	0.67	NA

Abbreviations: BMI: body mass index; CI: confidence interval; DL_CO_: diffusing capacity of the lung for carbon monoxide; FVC: forced vital capacity; MD: mean difference; NA: not available; OR: odds ratio.

^a^ *p* value for the estimate of effect.

^b^ *p* value for the Cochrane *Q* test.

^c^ *p* value for the Egger's test.

### 2.5 Lung Function of Patients with PRISm

According to the definition of PRISm, FEV_1_ and FEV_1_/FVC were not analyzed in the study. Pooled results of FVC and FVC% predicted demonstrated that PRISm population had both worse FVC (L) (MD: −0.78, 95%CI: −0.90 to −0.66) and FVC% predicted (MD: −24.74, 95%CI: −26.33 to −23.16) (Table 3). Egger's tests suggested the potential publication bias (FVC: *p* = 0.05; FVC% predicted: *p* = 0.02). However, the pooled results remained robust even after trim and fill analyses (Table S3).

### 2.6 Associated Comorbidities of PRISm

For cardiovascular diseases, the presence of PRISm was associated with a higher risk of overall cardiovascular disease (OR: 1.59, 95%CI: 1.31–1.92), hypertension (OR: 1.60, 95%CI: 1.29–2.00), ischemic heart disease (OR: 1.98, 95%CI: 1.71–2.30), heart failure (OR: 3.22, 95%CI: 1.90–5.47), and stroke (OR: 2.09, 95%CI: 1.78–2.44) (Table 3). For endocrine and metabolic diseases, PRISm was associated with an elevated risk of diabetes (OR: 1.94, 95%CI: 1.71–2.19), dyslipidemia (OR: 1.34, 95%CI: 1.17–1.54), and obesity or overweight (OR: 1.65, 95%CI: 1.16–2.34) (Table 3). Besides, PRISm was also related to respiratory comorbidities including chronic bronchitis (OR: 1.82, 95%CI: 1.47–2.24) and asthma (OR: 1.96, 95%CI: 1.77–2.17) (Table 3). Chronic kidney disease was more common in PRISm (OR: 1.66, 95%CI: 1.03–2.67) (Table 3), while no significant correlation was observed in tuberculosis, cancer, osteoporosis, anemia, or depression. Egger's tests indicated that publication bias might existed in the association of PRISm with heart failure and diabetes (heart failure: *p* = 0.03; diabetes: *p* < 0.01), while trim and fill analyses demonstrated that the pooled results were robust (Table S3).

## 3 Discussion

In this meta‐analysis, we demonstrated that the prevalence of PRISm is about 11% with potential risk factors including age, smoking status, and education. In addition, the FVC and FVC% predicted decreased in individuals with PRISm, while cardiovascular and endocrine comorbidities are also more common in PRISm. To the best of our knowledge, this is the first meta‐analysis comprehensively concluding the prevalence, risk factors, lung function, and comorbidities of PRISm, which may help identify and follow up high‐risk populations of PRISm in clinical settings.

Previous studies confirmed that PRISm is associated with COPD development, adverse cardiovascular or respiratory outcomes, and mortality [3, 4, 9]. Thus, GOLD has differentiated this phenotype from unclassified or nonspecific patterns, emphasizing the role of PRISm as a novel risk factor for health rather than just a subclinical spirometric phenotype, which could be detected and treated early [5]. Therefore, standard reporting of PRISm in routine lung function tests could be particularly important. Our findings partly supported the discrepancy between pre‐ and postbronchodilator PRISm, suggesting the procedure of bronchodilator tests may be needed to identify PRISm population. However, prebronchodilator spirometry might still be useful to some extent, especially for institutions not able to conduct postbronchodilator spirometry. Nevertheless, further confirmation of these positive findings using bronchodilators is still necessary for accurate diagnosis and to avoid overestimation.

Many studies revealed that the transition from normal lung function to PRISm and subsequently to COPD is a reversible trajectory, emphasizing the need to identify the population at high risk of persistent PRISm and even progression to COPD [6, 13, 16, 18, 21, 37, 42]. Potential interventions for these individuals, including early use of bronchodilators, pulmonary rehabilitation, and standardized management of comorbidities, may help prevent the progression or revert PRISm to normal. The studies by He and Miura provided novel insight into stratifying PRISm into two subtypes (with or without FVC reduction), which are heterogeneous in baseline characteristics and outcomes [32, 37]. Our study also revealed a decrease in FVC in PRISm population; thus, we hypothesized that FVC and FVC% predicted might be key factors affecting the natural history of PRISm. However, future evidence is needed.

Several studies also interpreted the roles of radiological features from computed tomography (CT) in patients with PRISm. For instance, quantitative analyses of CT in COPDGene cohorts demonstrated that individuals with PRISm have abnormal imaging parameters including increased airway wall thickness [29, 43], decreased total lung capacity (TLC), and emphysema [44, 45]. Other cohorts further confirmed these findings from COPDGene and indicated an elevated residual volume to TLC ratio in individuals with PRISm, emphasizing the potential air trapping despite decreased TLC [24, 46–48]. These radiological features were common in respiratory diseases such as chronic bronchitis and asthma, which were demonstrated as comorbidities of PRISm in our study. Besides, CT scans may also provide information beyond just lung disease, because the imaging abnormalities from chest CT could also help the detection of coronary artery calcification, ventricular remodeling, and osteoporosis, which were also identified as PRISm comorbidities in our findings [49]. According to this evidence, imaging features are important in the diagnosis and stratification of PRISm, and even the detection of comorbidities. Fortunately, the increased use of CT in lung cancer screening and health check‐ups brought novel opportunities to detect respiratory and nonrespiratory comorbidities at an early stage [50]. Therefore, synchronous lung function tests in these participants may bring clinical benefit, especially for PRISm and COPD, and future studies are needed to determine the effect of CT parameters on outcomes of PRISm in these participants.

A novel genome‐wide association study demonstrated the genetic association of PRISm with hypertension, diabetes, and myocardial infarction, which is consistent with our study [51]. As smoking is a common risk factor for respiratory, cardiovascular, and endocrine diseases, it might be a critical mediator contributing to the development of these comorbidities in individuals with PRISm. Therefore, the impact of smoking on comorbidities and outcomes of PRISm should be prioritized in future studies.

There are several limitations in our study. First, a majority of included studies were retrospective or secondary analyses with high heterogeneity even after conducting subgroup analysis and meta‐regression to find the origin. However, their study design was rigorous and follow‐up was adequate, resulting in a high NOS score, which did not attenuate the quality or credibility of pooled results. Second, the UK Biobank and Taiwan cohort accounted for most of the total population [16, 40], while the sample sizes of some studies were small. Nevertheless, we conducted sensitivity analyses to detect the overrepresentation of each study, and the results supported the robustness of pooled results. Besides, Egger's tests were performed to detect the small sample size effects and publication bias, which did not exist for most associations. Third, some potential risk factors (including diet, alcohol intake, and social‐economical levels) and other lung function parameters were not analyzed due to a lack of data, suggesting the importance of future validation. Fourth, the effect direction of age and sex altered after trim and fill analyses, leading to difficulty in interpretation. We hypothesized that these results may be confounded by other factors like smoking status and must be considered more rigorously; however, it is hoped that future evidence could be constructed to resolve this issue and validate the true effect of age and sex on PRISm risk.

## 4 Conclusions

Taken together, our findings shed light on the prevalence, risk factors, lung function, and associated comorbidities of adult PRISm. The diagnosis and stratification of PRISm requires comprehensive consideration of clinical characteristics, lung function, and radiological features. Therefore, future studies exploring the mechanisms, radiological features, and subtypes of PRISm are needed to provide a deeper understanding of this disease for clinicians, and ultimately improve the clinical outcomes of patients.

## 5 Materials and Methods

### 5.1 Reporting Guidelines and Registration

This study was conducted following the Preferred Reporting Items for Systematic Review and Meta‐analyses (PRISMA) guideline [52] and registered on the International Prospective Register of Systematic Reviews (PROSPERO) (number CRD42024526075).

### 5.2 Search Strategies

Potential publications were searched in Medline, Web of Science, Embase, and Scopus up to March 26, 2024, using keywords including “preserved ratio impaired spirometry,” “pre‐COPD,” “early COPD,” “GOLD‐Unclassified,” and so on. Full search strategies for all databases are available in Table S4.

### 5.3 Definition of PRISm

According to previous studies, both the fixed ratio and LLN definitions of PRISm were accepted in this study [4]. The fixed ratio was defined as an FEV_1_/FVC ≥ 0.7 with an FEV_1_% predicted <0.8, either before or after use of bronchodilators. The LLN definition was an FEV_1_/FVC ≥ LLN with an FEV_1_% predicted < LLN, while thresholds of LLN definition were based on LLN or *Z*‐score cutoff points in the study population, either before or after use of bronchodilators. If prebronchodilator and postbronchodilator analyses were both provided, postbronchodilator results were preferred.

### 5.4 Eligibility Criteria

The inclusion criteria were as follows: (1) observational studies of general population or lung cancer screening cohorts, including cohort studies, case–control studies, and cross‐sectional studies; (2) study population over 18 years old; (3) pre‐ or postbronchodilator spirometry conducted; (4) studies reporting the prevalence of PRISm; and (5) original research with full‐text available.

The exclusion criteria were as follows: (1) lack of data; (2) wrong definition of PRISm; (3) nonadult; (4) nonrepresentative population (e.g., all from outpatients or with certain diseases); (5) studies based on overlapped or duplicate population, wherein the most comprehensive study was prioritized.

### 5.5 Literature Selection

Two researchers (H. Wang and R. Yang) independently selected the literature and discrepancies were resolved by team consensus. The titles and abstracts of records identified in databases were initially screened to remove duplication and irrelevant studies. Potential publications were comprehensively reviewed in full text.

### 5.6 Data Extraction

Two researchers (H. Wang and R. Yang) extracted the following variables from the included studies independently: the first author, publication year, name of the study or cohort, study region, population, sample size, number of PRISm cases, definition of PRISm, pre‐ or postbronchodilator, age, sex, BMI, smoking status, education, lung function, and comorbidities (including cardiovascular diseases, endocrine or metabolic diseases, pulmonary diseases, kidney diseases, cancer, mental disorders, etc.). Continuous variables were extracted in forms of mean and standard deviation, and data were estimated using a previously published method if only medians with ranges or interquartile ranges were available [53]. Any disagreement was resolved by team discussion to reach a consensus.

### 5.7 Quality Assessment

We used the Newcastle‐Ottawa Quality Assessment Scale (NOS) to assess the quality of included studies [54]. The NOS comprises three domains: selection, comparability, and outcome, each contributing 4, 2, and 3 points, respectively. High‐quality studies were defined as studies labeled with more than 6 points.

### 5.8 Statistical Analyses

The random effects model was used to merge the results, and only associations including more than two studies were analyzed. Pooled prevalence was estimated with a corresponding 95% confidence interval (CI) using the Freeman–Tukey double arcsine transformation and inverse variance method [55]. Mean differences (MDs) were pooled to describe continuous variables, while the Mantel–Haenszel method was used to pool the results of binary variables with odds ratios (ORs). Heterogeneity among the included studies was evaluated by *I* ^2^ statistics and Cochrane *Q* tests, and significant heterogeneity was defined as an *I* ^2^ > 50% or *p* value < 0.10 for Cochrane *Q* test. Subgroup and sensitivity analyses based on the leave‐one‐out method or certain confounders such as sex, smoking status, study population, PRISm definition, pre‐ or postbronchodilator, study region, sample size, and NOS were conducted to detect the robustness of results and the source of heterogeneity. Publication bias was evaluated by funnel plot and Egger's test, and a trim and fill analysis was performed to adjust the results if obvious publication bias existed [56, 57]. *p* Values < 0.05 were regarded as statistically significant except for the results of Cochrane *Q* tests. All statistical analyses and visualizations were conducted using R (version 4.2.2, R Foundation for Statistical Computing, Vienna, Austria) with the “meta” package (version 6.2.1)

## Author Contributions

Haoyu Wang: methodology, project administration, software, validation, formal analysis, investigation, resources, data curation, visualization, writing—original draft; Ruiyuan Yang: validation, data curation, writing—review and editing; Dan Liu: conceptualization, supervision, writing—review and editing; Weimin Li: conceptualization, supervision, funding acquisition, writing—review and editing. All authors have read and approved the final manuscript.

## Ethics Statement

The authors have nothing to report.

## Conflicts of Interest

The authors declare no conflicts of interest.

## Supporting information



Supporting Information

## Data Availability

All data analyzed in the study were previously published and can be accessed from the respective journals.
