# Supplementary material for: Prevalence, Risk Factors, Lung Function, and Associated Comorbidities of Adult Preserved Ratio Impaired Spirometry: A Meta‐Analysis
Source: MedComm (2020). 2025 May 31;6(6):e70235. doi: 10.1002/mco2.70235 (PMC12126599; doi:10.1002/mco2.70235)
Supplement: Supplementary file 1 — Supporting Information [file MCO2-6-e70235-s001.pdf]

Study	IV, Random, 95% CI
Omitting Wijnant 2020	0.11 [0.10, 0.13]
Omitting Anami 2021	0.11 [0.10, 0.13]
Omitting Kaise 2021	0.11 [0.09, 0.13]
Omitting Marott 2021	0.11 [0.09, 0.12]
Omitting Schwartz 2021	0.11 [0.09, 0.13]
Omitting Wan 2021	0.11 [0.10, 0.13]
Omitting Higbee 2022	0.11 [0.09, 0.13]
Omitting Kaaks 2022	0.11 [0.09, 0.13]
Omitting Kanetake 2022	0.11 [0.10, 0.13]
Omitting Kim 2022	0.11 [0.10, 0.13]
Omitting Shiraishi 2022	0.11 [0.10, 0.13]
Omitting Tanabe 2022	0.11 [0.10, 0.13]
Omitting Tang 2022	0.11 [0.09, 0.13]
Omitting Washio 2022	0.11 [0.10, 0.13]
Omitting Zhao 2022	0.11 [0.10, 0.13]
Omitting Chen 2023	0.11 [0.10, 0.13]
Omitting Fan 2023	0.11 [0.10, 0.13]
Omitting Kogo 2023	0.11 [0.10, 0.13]
Omitting Krishnan 2023	0.11 [0.10, 0.13]
Omitting Labaki 2023	0.11 [0.10, 0.13]
Omitting Magner 2023	0.11 [0.10, 0.13]
Omitting Marott 2023	0.11 [0.10, 0.13]
Omitting Miura 2023	0.11 [0.10, 0.13]
Omitting Perez−Padilla 2023	0.11 [0.10, 0.13]
Omitting Sin 2023	0.11 [0.10, 0.13]
Omitting Zhang 2023	0.11 [0.09, 0.13]
Omitting Choi 2024	0.11 [0.09, 0.13]
Omitting He 2024	0.11 [0.10, 0.13]
Omitting Im 2024	0.11 [0.09, 0.12]
Omitting Lei 2024	0.11 [0.10, 0.13]
Omitting Shu 2024	0.11 [0.09, 0.13]
Omitting Zhang 2024	0.11 [0.09, 0.12]
**Total (95% CI)**	**0.11 [0.10, 0.13]**

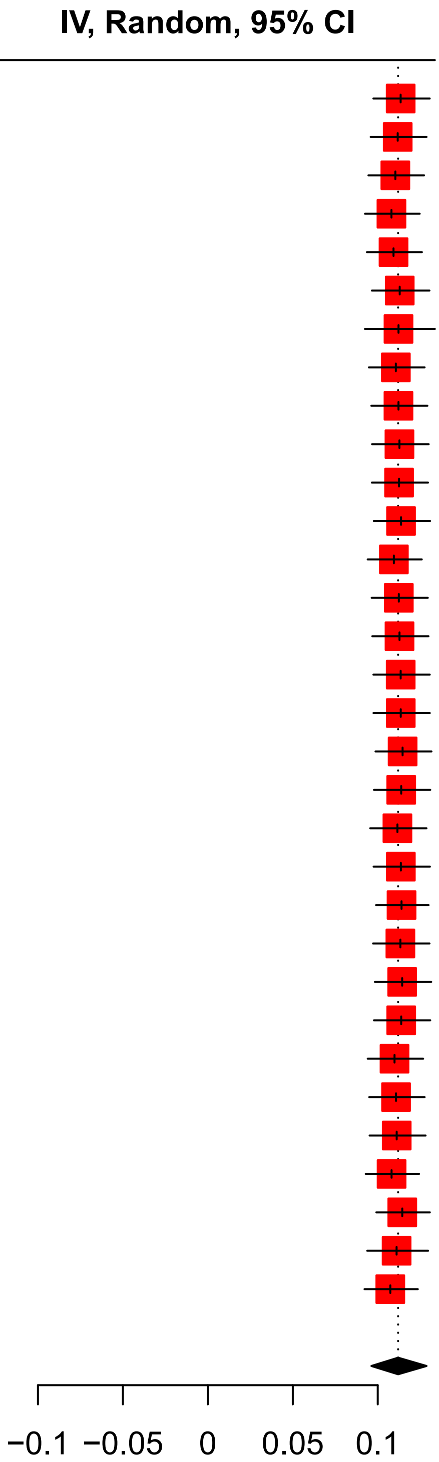

Figure S1. Leave-one-out sensitivity analysis of the prevalence of PRISm.
IV: inverse variance; CI: confidence interval.

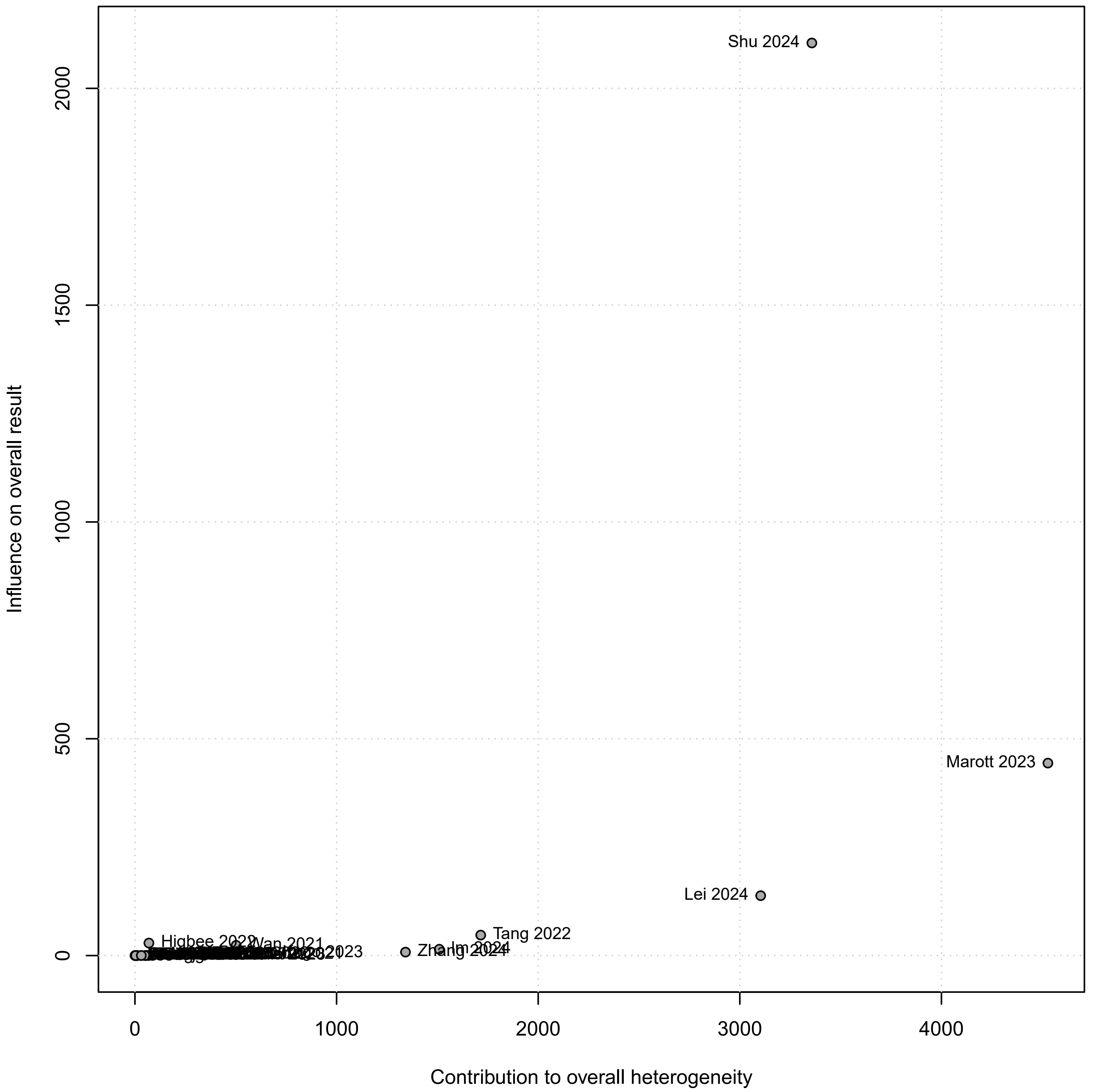

Figure S2. Baujat plot of the prevalence of PRISm.

Table S1. Results of quality assessment of each study using NOS

Assessment criteria	Selection				Comparability		Outcome			Total
	Representativeness of the exposed cohort	Selection of the nonexposed cohort	Ascertainment of exposure	Outcome not present at start of study	Comparability based on design or analysis		Assessment of outcome	Enough follow-up	Adequacy of follow up	
Wijnant 2020	●	●	●	●	●	○	●	●	○	**7**
Anami 2021	●	●	●	●	●	●	●	○	○	**7**
Kaise 2021	●	●	●	●	●	○	●	○	○	**6**
Marott 2021	●	○	●	●	●	○	●	●	●	**7**
Schwartz 2021	●	●	●	●	●	○	●	○	○	**6**
Wan 2021	●	●	●	●	●	○	●	●	●	**8**
Higbee 2022	●	●	●	●	●	○	●	●	●	**8**
Kaaks 2022	●	●	●	●	●	○	●	●	●	**8**
Kanetake 2022	●	●	●	●	●	○	●	●	●	**8**
Kim 2022	●	●	●	●	●	○	●	●	●	**8**
Shiraishi 2022	●	●	●	●	●	○	●	●	○	**7**
Tanabe 2022	●	●	●	●	●	○	●	●	●	**8**
Tang 2022	●	●	●	●	●	○	●	○	○	**6**
Washio 2022	●	●	●	●	●	○	●	●	●	**8**
Zhao 2022	●	●	●	●	●	○	●	○	○	**6**
Chen 2023	●	●	●	●	●	○	●	●	●	**8**
Fan 2023	●	●	●	●	●	○	●	●	●	**8**
Kogo 2023	●	●	●	●	●	●	●	●	●	**9**
Krishnan 2023	●	●	●	●	●	○	●	●	●	**8**
Labaki 2023	●	●	●	●	●	○	●	●	●	**8**
Magner 2023	●	●	●	●	○	○	●	●	○	**6**
Marott 2023	●	●	●	●	●	○	●	●	●	**8**
Miura 2023	●	●	●	●	●	○	●	○	○	**6**
Perez-Padilla 2023	●	●	●	●	●	○	●	●	●	**8**
Sin 2023	●	●	●	●	○	○	●	●	●	**7**
Zhang 2023	●	●	●	●	●	○	●	○	●	**7**
Choi 2024	●	●	●	●	●	●	●	●	○	**8**

He 2024	●	●	●	●	○	○	●	●	●	**7**
lm 2024	●	●	●	●	●	○	●	●	○	**7**
Lei 2024	●	●	●	●	●	○	●	○	○	**6**
Shu 2024	●	●	●	●	●	○	●	●	●	**8**
Zhang 2024	●	●	●	●	●	○	●	●	○	**7**

Abbreviations: NOS: Newcastle-Ottawa Quality Assessment Scale.

Table S2. Subgroup analyses of the prevalence of PRISm.

Subgroup	Number of studies	Prevalence (95%CI)	I^2 (%)	$p_{Cochrane}$[a]	$p_{Difference}$[b]
Sex					0.75
Male	29	11% (10% to 13%)	100	<0.01	
Female	29	11% (9% to 13%)	100	<0.01	
Smoking status					0.10
Never smoker	24	10% (8% to 12%)	100	<0.01	
Ever smoker	25	11% (10% to 13%)	100	<0.01	
Current smoker	25	13% (11% to 14%)	99	<0.01	
Definition					0.56
Fixed ratio	28	11% (10% to 13%)	100	<0.01	
LLN	4	10% (4% to 16%)	100	<0.01	
Pre/Post-BD					0.06
Pre-BD	21	12% (10% to 14%)	100	<0.01	
Post-BD	9	8% (6% to 11%)	99	<0.01	
NR	2	13% (5% to 24%)	99	<0.01	
Region					0.31
Europe	6	12% (9% to 16%)	100	<0.01	
Asia	19	12% (9% to 15%)	100	<0.01	
America	7	9% (6% to 12%)	99	<0.01	
Sample size					0.70
≥10000	11	11% (8% to 13%)	100	<0.01	
<10000	21	11% (9% to 15%)	99	<0.01	
NOS					0.04
8-9	16	9% (7% to 11%)	100	<0.01	
6-7	16	14% (9% to 19%)	100	<0.01	

Abbreviations: PRISm: preserved ratio impaired spirometry; CI: confidence interval; LLN: lower limit of normal; BD: bronchodilator; NR: not reported.

a: p-value for the Cochrane Q test

b: p-value for the subgroup difference

Table S3. Trim and fill analyses.

	p_{Egger}	Adjusted estimate (95%CI)	p
Age (years)	<0.01	-0.36 (-0.86 to 0.13)	0.15
Sex (male)	0.01	0.89 (0.81 to 0.98)	0.01
FVC (L)	0.05	-0.63 (-0.74 to -0.52)	<0.01
FVC % predicted	0.02	-20.24 (-21.73 to -18.75)	<0.01
Heart failure	0.03	1.92 (1.21 to 3.05)	<0.01
Diabetes	<0.01	2.58 (2.28 to 2.92)	<0.01

Abbreviations: CI: confidence interval; FVC: forced vital capacity.

Table S4. Search strategies

**Date: March 26, 2024**	
**Medline**	
1	preserved ratio impaired spirometry
2	pre-COPD
3	"early chronic obstructive pulmonary disease" OR "early COPD"
4	GOLD-Unclassified OR GOLD-U
5	"restrictive spirometr*" OR "impaired spirometr*" OR "abnormal spirometr*"
6	"spirometr* restriction" OR "spirometr* abnormality" OR "spirometr* impairment"
7	"restrictive lung function" OR "restrictive pulmonary function"
8	#1 OR #2 OR #3 OR #4 OR #5 OR #6 OR #7
**Web of Science**	
1	TS=(preserved ratio impaired spirometry)
2	TS=(pre-COPD)
3	TS=("early chronic obstructive pulmonary disease" OR "early COPD")
4	TS=(GOLD-Unclassified OR GOLD-U)
5	TS=("restrictive spirometr*" OR "impaired spirometr*" OR "abnormal spirometr*")
6	TS=("spirometr* restriction" OR "spirometr* abnormality" OR "spirometr* impairment")
7	TS=("restrictive lung function" OR "restrictive pulmonary function")
8	#8: #1 OR #2 OR #3 OR #4 OR #5 OR #6 OR #7
**Embase**	
1	preserved ratio impaired spirometry.mp.
2	pre-COPD.mp.
3	(early chronic obstructive pulmonary disease or early COPD).mp.
4	(GOLD-Unclassified or GOLD-U).mp.
5	(restrictive spirometr* or impaired spirometr* or abnormal spirometr*).mp.
6	(spirometr* restriction or spirometr* abnormality or spirometr* impairment).mp.
7	(restrictive lung function or restrictive pulmonary function).mp.
8	1 or 2 or 3 or 4 or 5 or 6 or 7
**Scopus**	
1	ALL("preserved ratio impaired spirometry" OR "pre-COPD" OR "early chronic obstructive pulmonary disease" OR "early COPD" OR "GOLD-Unclassified" OR "GOLD-U" OR "restrictive spirometry" OR "impaired spirometry" OR "abnormal spirometry" OR "spirometric restriction" OR "spirometric abnormality" OR "spirometric impairment" OR "restrictive lung function" OR "restrictive pulmonary function")
